# Development and characterization of a dedicated dose monitor for ultrahigh-dose-rate scanned carbon-ion beams

**DOI:** 10.1038/s41598-024-62148-2

**Published:** 2024-05-21

**Authors:** Masashi Yagi, Shinichi Shimizu, Noriaki Hamatani, Takuto Miyoshi, Takuya Nomura, Takashi Toyoda, Mahoro Nakatani, Toshiro Tsubouchi, Masaki Shimizu, Yoshiaki Kuwana, Masumi Umezawa, Masaaki Takashina, Teiji Nishio, Masahiko Koizumi, Kazuhiko Ogawa, Tatsuaki Kanai

**Affiliations:** 1 Department of Carbon Ion Radiotherapy, Osaka University Graduate School of Medicine, Osaka, Japan; 2 Department of Medical Physics, Osaka Heavy Ion Therapy Center, Osaka, Japan; 3 Hitachi, Ltd. Research & Development Group, Ibaraki, Japan; 4 Healthcare Business Division, Hitachi, Ltd, Chiba, Japan; 5 Medical Physics Laboratory, Division of Health Science, Osaka University Graduate School of Medicine, Osaka, Japan; 6 Department of Medical Physics and Engineering, Graduate School of Medicine, Osaka, Japan; 7 Department of Radiation Oncology, Osaka University Graduate School of Medicine, Osaka, Japan; 8 Healthcare Business Groupe, Hitachi High-Tech Corporation, Chiba, Japan; 9 Department of Radiation Oncology, Nozaki Tokushukai Hospital, Osaka, Japan

**Keywords:** Applied physics, Radiotherapy

## Abstract

The current monochromatic beam mode (i.e., uHDR irradiation mode) of the scanned carbon-ion beam lacks a dedicated dose monitor, making the beam control challenging. We developed and characterized a dedicated dose monitor for uHDR-scanned carbon-ion beams. Furthermore, a simple measurable dose rate (dose rate per spot (DR_spot_)) was suggested by using the developed dose monitor and experimentally validating quantities relevant to the uHDR scanned carbon-ion beam. A large plane-parallel ionization chamber (IC) with a smaller electrode spacing was used to reduce uHDR recombination effects, and a dedicated operational amplifier was manufactured for the uHDR-scanned carbon-ion beam. The dose linearity of the IC was within ± 1% in the range of 1.8–12.3 Gy. The spatial inhomogeneity of the dose response of the IC was ± 0.38% inside the ± 40-mm detector area, and a systematic deviation of approximately 2% was measured at the edge of the detector. uHDR irradiation with beam scanning was tested and verified for different doses at the corresponding dose rates (in terms of both the average dose rate and DR_spot_). We confirmed that the dose monitor can highlight the characteristics (i.e., dose, dose rate, and dose profile) of uHDR-scanned carbon-ion beams at several dose levels in the monochromatic beam mode.

## Introduction

The FLASH effect efficiently inhibits tumor growth to the same degree as the currently employed conventional dose rate (typically in cGy/s) while minimizing damage to healthy tissues. Ultrahigh-dose-rate (uHDR) radiation with photon^1^, electron^2^ and proton^3^ beams can potentially increase the therapeutic window between the tumor control rate and normal tissue toxicity. uHDR beams have been applied to humans to demonstrate the feasibility of FLASH radiotherapy^4,5^. In addition, recent studies suggested that the uHDR irradiations may act differently on tumors compared to normal dose rate^6–8^.

Few reports exist on the in vivo and in vitro use of carbon-ion beams scanned at an uHDR. uHDR carbon-ion beams applied at a dose and dose rate of 7.4 Gy and 40 Gy/s, respectively, induced normal tissue sparing with brain organoids at a relevant plateau linear energy transfer (LET) level of 12 keV/μm^9^. The sparing effect was observed in case of Chinese hamster ovary cells (CHO-K1) using a carbon-ion beam applied at a dose and dose rate of 7.4 Gy and 70 Gy/s, respectively, and with a dose-averaged LET of 13 keV/μm in hypoxia^10^. Furthermore, the FLASH effect was observed for C3H/He mice osteosarcoma in the hind limb with a dose of 18 Gy at 100 Gy/s with ~ 15 keV/μm LET^6^. However, the sparing effect was not observed under aerobic conditions in HFL1 and HSGc-C5 cells with doses of 1–3 Gy at 96–195 Gy/s with 13- or 50-keV/μm LET^11^. Although several mechanisms of the FLASH effect in carbon-ion beams have been postulated^12^, the general action mechanism remains unclear. Verifying the FLASH effect using carbon ions will contribute to its better understanding of the FLASH effect as well as its LET dependence.

Few systems available worldwide can conduct uHDR-scanned carbon-ion research although carbon-ion beam-related requirements must be urgently identified for uHDR research^12^. We previously developed the monochromatic beam mode^13,14^ for the HyBeat Heavy-ion Therapy System (Hitachi, Ltd., Tokyo, Japan)^15^ for uHDR irradiation. The monochromatic beam mode was designed to acquire a pristine monochromatic beam by avoiding passage through the nozzle. The spill length and beam current were set to run at preprogrammed values.

A major challenge for performing uHDR irradiation with carbon-ions is beam monitoring, which is used to control the scanned beam and dosimetry. Accurate and reliable dosimetry is critical for the applications of uHDR scanned carbon-ion beams. Typically, a plane-parallel ionization chamber (IC) is utilized as the dose monitor for carbon-ion beams. A plane-parallel IC continuously monitors the particle flux of the beam to control the raster scanning process during irradiation in the monitor unit (MU)^12^. If the desired number of particles, defined by the treatment planning system, is reached, the scanning magnets direct the beam to the next position. The current monochromatic beam mode does not have a dose monitor and cannot control the beam in the MU because it avoids the passage of the beam through the nozzle. In addition, a dedicated dose monitor is required for the uHDR beam to apply beam currents larger than the conventional-dose-rate beam. To enable accurate beam monitoring using the monochromatic beam mode, we developed and characterized a dose monitor for uHDR-scanned carbon-ion irradiation.

In addition, the experimental validation of quantities relevant to the uHDR carbon-ion beam remain lacking. The uHDR carbon-ion beam requires monitoring the dwell time and the delivered dose, which are critical inputs for the experimental validation of the dose rate. The dose rate is currently investigated by employing simulations^16^. The scanning delivery introduces a unique spatiotemporal correlation of the delivered radiation, complicating the definition and characterization of the dose rate. Therefore, a measurable simple dose-rate was suggested by utilizing the developed dose monitor in this study.

## Results

### Characterization of the operational amplifier

**Figure 1:**
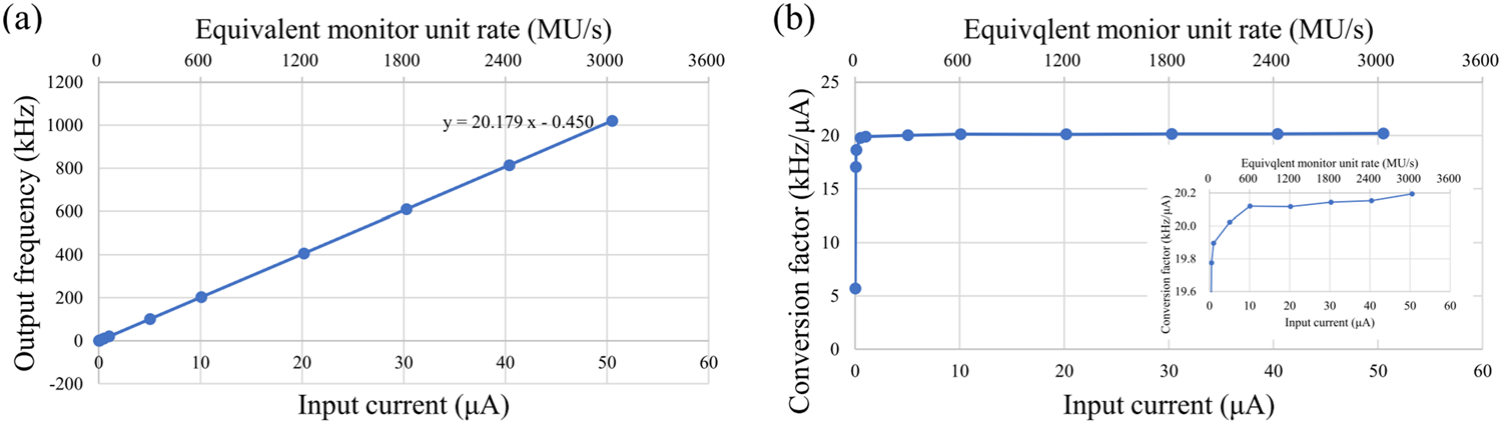
Linearity of the operational amplifier. The linearity of the output frequency as a function of input current was within ± 1% in the examined range. The plot of (**a**) output frequency and (**b**) conversion factor against the input current. The upper horizontal axis corresponds to the estimated equivalent MU rate. The equivalent MU rate was calculated from the output frequency assuming that the IC was connected. The absolute values of the input current are used for display purposes.

Linearity was observed between the input current and the output frequency (Fig. 1). The linearity of the output frequency as a function of input current was within ± 1% in the range of − 0.5– − 50 μA (equivalent MU rate: 33.7–3.367 MU/s and beam current: 1.05–105 nA at 208.3 MeV/u). The equivalent MU rate was calculated from the output frequency assuming that the IC was connected.

### Characterization of the IC

**Figure 2:**
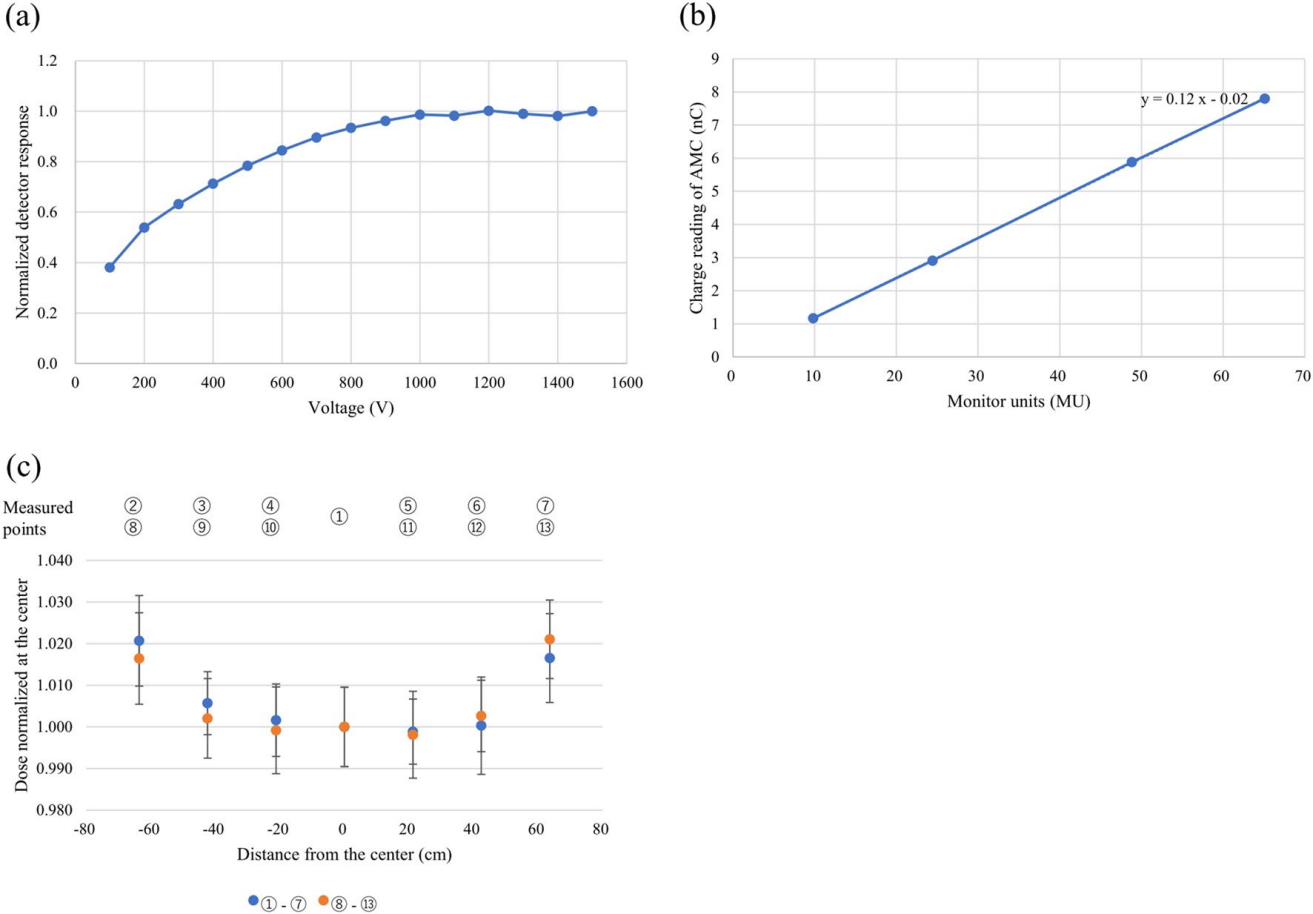
Characterization of the IC for uHDR irradiations: (**a**) saturation measurements of the ion collection efficiency with varying voltages of the IC under uHDR conditions. The absolute values of the voltage are used for display purposes. (**b**) Dose linearity of the IC obtained using the uHDR carbon-ion beam (208.3 MeV/u). The plot of the dose against the input MU. (**c**) Spatial inhomogeneity of the dose response of the IC.

The saturation curve of the ion collection efficiency for uHDR irradiations is shown in Fig. 2a. The detector response was normalized based on the value at − 1500 V. The IC saturation curve of the ion collection efficiency reached a plateau at − 1000 V, corresponding to an electric field strength of 5 kV/cm.

Figure 2b demonstrates the linearity in the dose of the IC in the examined MUs. The linearity in dose was within ± 1% in the range of 9.8 to 65.1 MU.

The inhomogeneity of the dose response of the IC was ± 0.38% within the ± 40 mm detector area, and a systematic deviation of approximately 2% was measured at the edge of the detector (Fig. 2c).

### uHDR application

**Figure 3:**
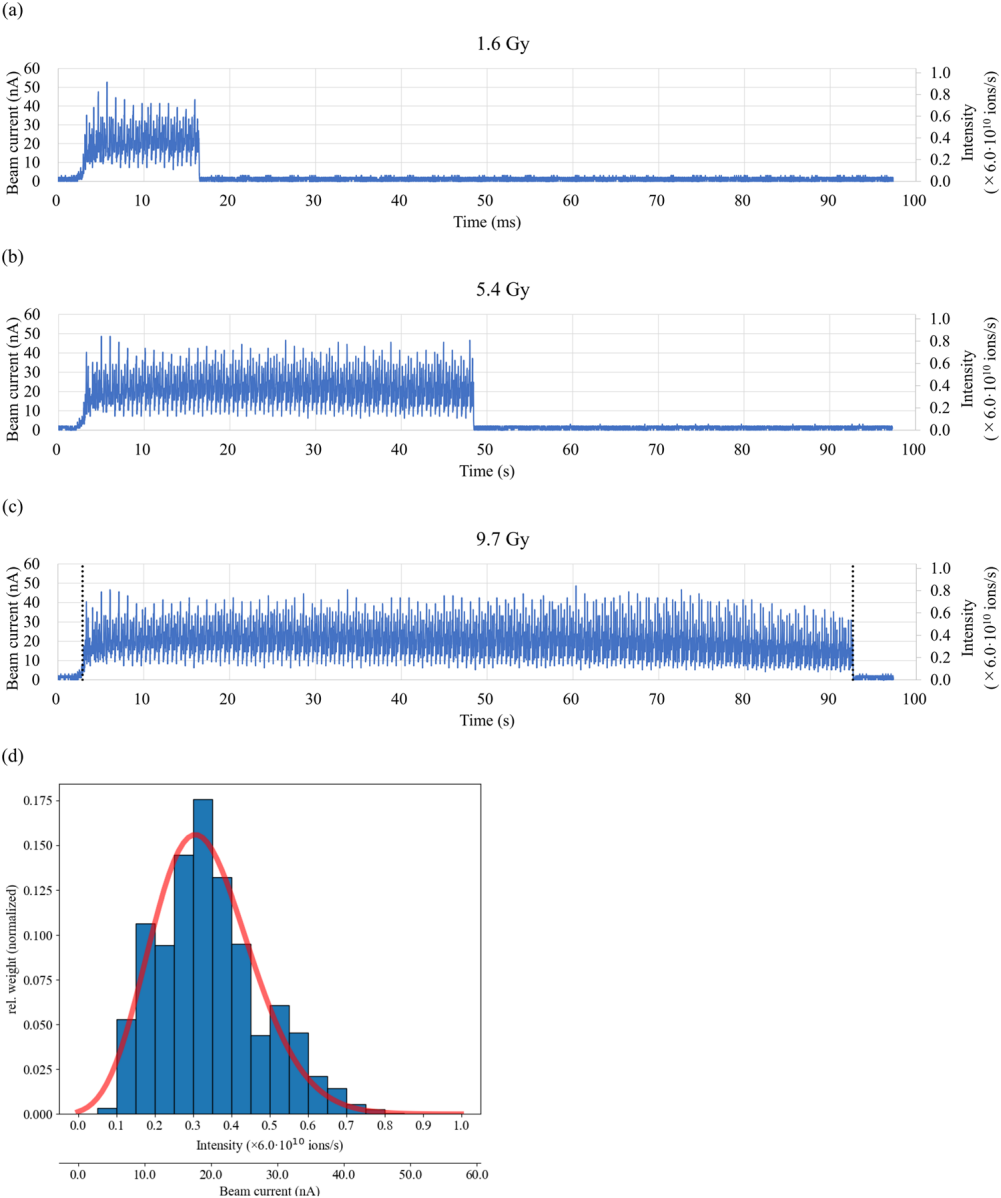
Measured beam current of the extracted beam as a function of time. The uHDR extraction with doses of (**a**) 1.6, (**b**) 5.4, and (**c**) 9.7 Gy. (**d**) Histogram between the black dashed lines shown in (**c**) for an uHDR irradiation at 9.7 Gy. The intensities were assigned to 20 different bins. The height of the bins corresponded to the relative duration of the particle fluence with these intensities during the spill. The red line shows the fitted curve with a Poisson distribution.

Figure 3 shows the beam intensity with a fine structure (ripple) at different dose levels, which is typical for a synchrotron extraction. The structure was characterized using a histogram, fitted by a Poisson distribution (the Poisson parameter λ was 0.331, corresponding to 1.989 × 10^10^ ions/s (= 0.331 × 6.0 × 10^10^ ions/s)).

**Figure 4:**
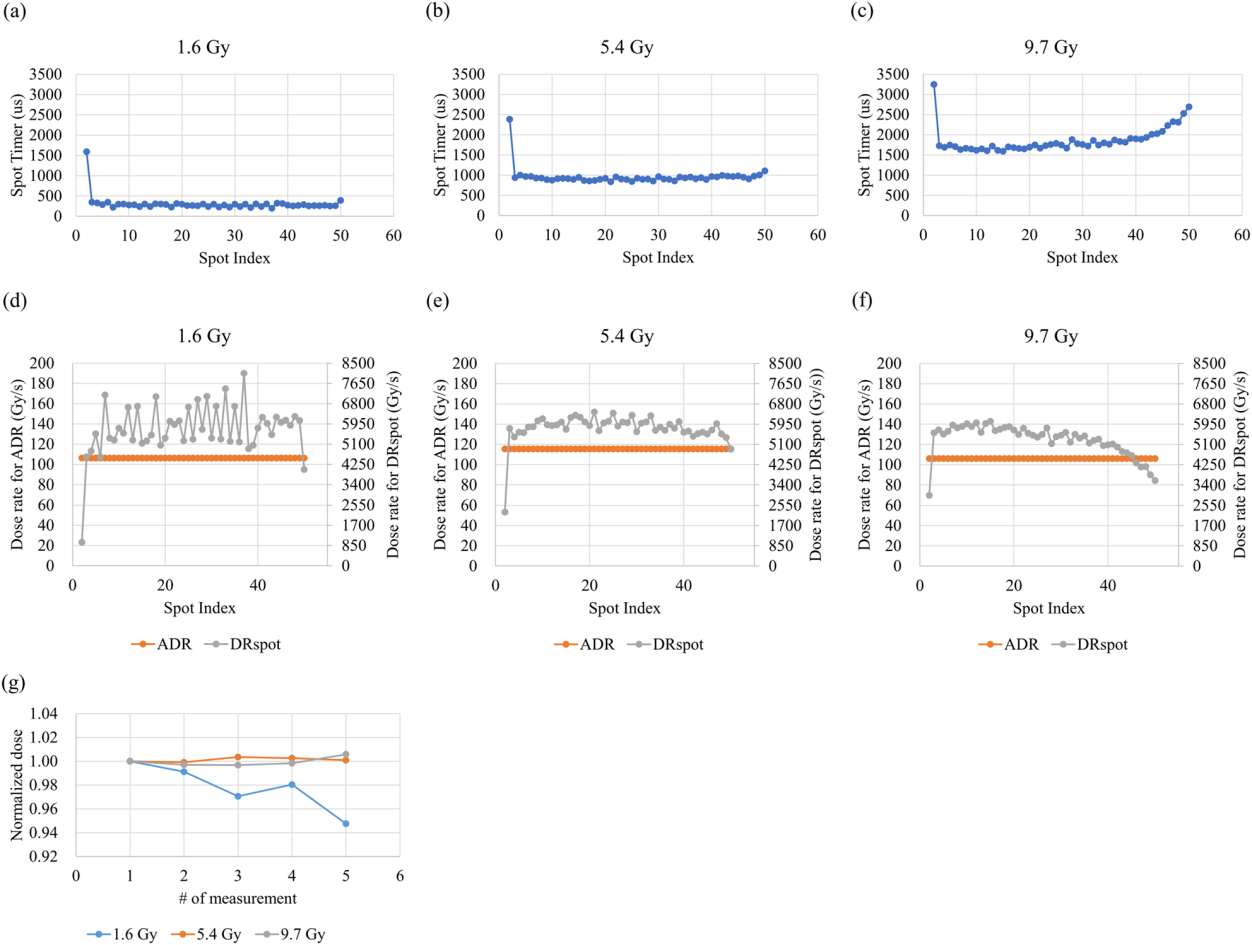
Characterized uHDR scanned carbon-ion beam with the developed IC. The spot timer against the spot index at (**a**) 1.6, (**b**) 5.4, and (**c**) 9.7 Gy. ADR and DR_spot_ against the spot index for (**d**) 1.6, (**e**) 5.4, and (**f**) 9.7 Gy. (**g**) Doses of uHDR irradiations for a series of tests (same plan file for a given dose) conducted in a day.

The irradiation time of the first spot was longer than those of the other spots (Fig. 4a–c). Particularly at 9.7 Gy, the irradiation times of the latter spots were also quite long (Fig. 4c). The fluctuation in DR_spot_ was large at 1.6 Gy and small at 9.7 Gy, whereas a large difference from the average dose rate (ADR) was observed in the latter spots. The accurate dose delivery of the uHDR irradiation with beam scanning was validated at 1.6 ± 0.03, 5.4 ± 0.01, and 9.7 ± 0.04 Gy (Fig. 4g) for the beam in an area of at least 16 mm × 16 mm at the dose rates of 106.0 ± 3.5, 114.2 ± 1.1, and 106.5 ± 5.5 Gy/s, respectively, in terms of the ADR (orange solid lines in Fig. 4d − f, respectively). The means and standard deviations of the dose rates (DR_spot_) at 1.6, 5.4, and 9.7 Gy were 5727.8 ± 1071.3, 5786.4 ± 595.6, and 5302.5 ± 663.4 Gy/s, respectively (gray solid lines in Fig. 4d − f). The difference between *MU* (plan) and *MU*_*i*_ (record) was less than 0.3%.

**Figure 5:**
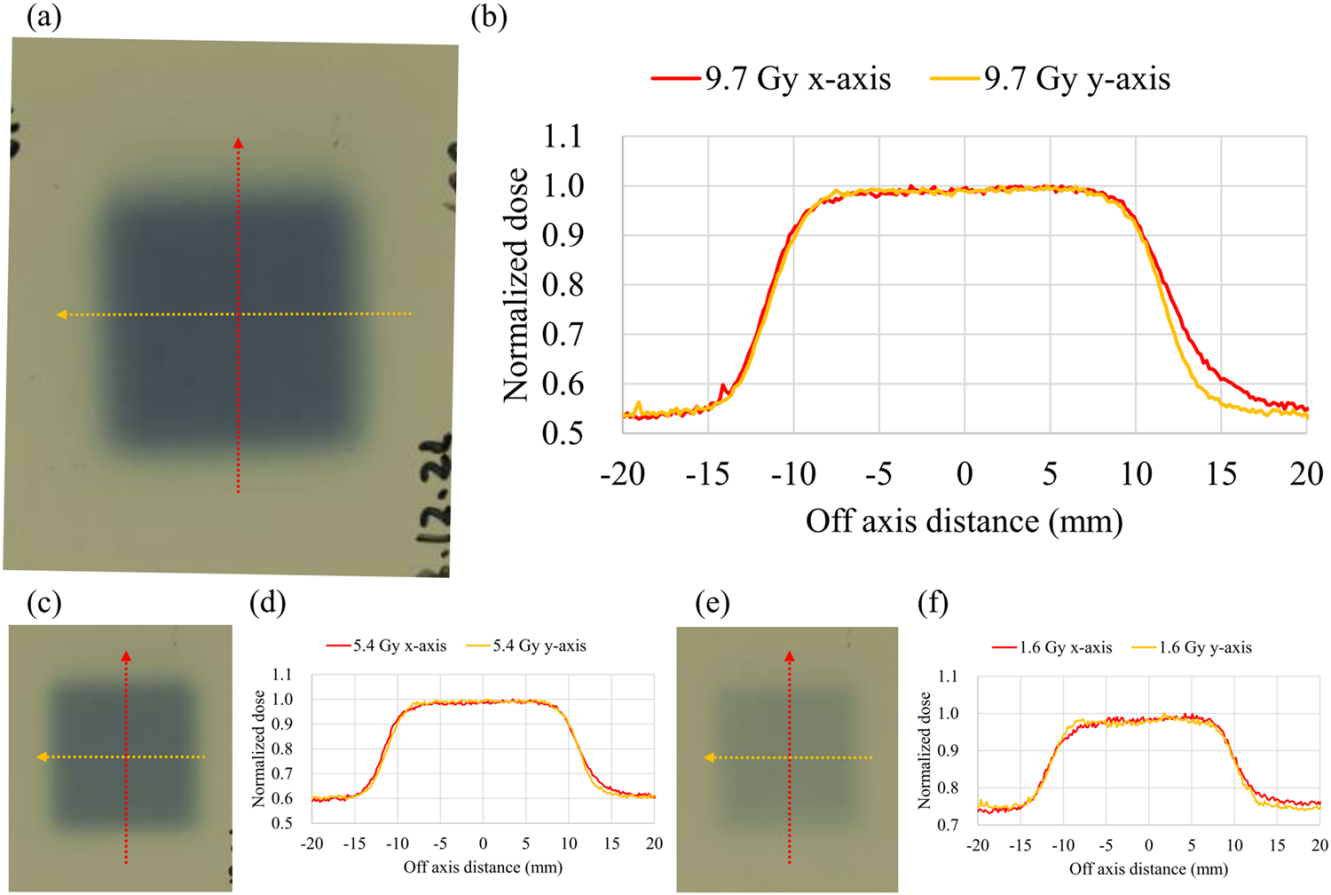
Measured lateral dose distributions for uHDR carbon-ion irradiations using the developed IC at 208.3 MeV/u for doses of 9.7 (**a** and **b**), 5.4 (**c** and **d**), and 1.6 Gy (**e** and **f**). The left and right pictures show the Gafchromic (EBT3) film measurement and the measured lateral dose profiles in the x and y directions for each panel. In the left picture, the dashed lines indicate the section for the profiles in the measured film. In the right picture, lateral dose profiles (x and y directions in red and yellow, respectively) for uHDR irradiations are shown. The axes are the beam definitions.

Figure 5 shows the scanned film image and lateral dose profiles at the isocenter. The dose profiles were normalized based on the maximum value. The flatness of the uHDR irradiation profile at 1.6, 5.4, and 9.7 Gy is indicated at approximately 2.5%. Table 1 shows a summary of the results.

**Table 1:** Dosimetry parameters of the measured dose distributions using the developed IC at ultrahigh dose rates.

	Flatness (%)		Absolute-dose measurement (Gy)	Dose rate (Gy/s, ADR)	Dose rate (Gy/s, DR_spot_)
1	X	1.7	9.67 ± 0.04	106.46 ± 5.53	5302.50 ± 663.44
	Y	1.3			
2	X	2.1	5.38 ± 0.01	114.23 ± 1.15	5786.38 ± 595.59
	Y	1.5			
3	X	1.9	1.57 ± 0.03	106.01 ± 3.45	5727.83 ± 1071.27
	Y	2.5			

## Discussion

The beam current ripple (Fig. 3a −c) was larger than that obtained from a beam employed at a conventional dose rate because the beam adjustment for uHDR irradiation requires increased beam current. The linearity of the output frequency as a function of input current for the developed operational amplifier was confirmed (within ± 1%) in the estimated current range of the uHDR beam (Fig. 1). The operational amplifier could be employed for measuring ionization currents from the dose monitor applied for the uHDR beam. As shown in Fig. 2, the IC saturation curve of the ion collection efficiency reached a plateau at − 1000 V. The repeatability of the absolute-dose measurement with the advanced Markus chamber (AMC) was within 0.4%, except for the ~ 2% value obtained at 1.6 Gy as a coefficient of variation calculated from Table 1, indicating that the IC could monitor the dose of the uHDR scanned carbon-ion beam under the beam intensity structure (Fig. 3). The beam intensity structure can be quantitively characterized by fitting the curve with a Poisson distribution (Fig. 3d). This characterization could be one of the experimental characterization and validation methods of quantities relevant for the uHDR carbon-ion. The fluctuation of the beam intensity at the lower dose per spot was not averaged because of the relatively short irradiation time per spot. The dose linearity of the IC connected to the operational amplifier for uHDR irradiation was confirmed (within ± 1%) in the examined dose range. The inhomogeneity of the dose response of the IC was ± 0.38% within ± 40 mm detector area while the systematic deviation of approximately 2% was measured at the edge of the detector. The distance between the electrodes could change owing to sagging due to the attraction in the electric field (i.e., the distance between the anode and the cathode was smaller at the center compared to those at the edges). In the calculation, a decrease of 0.05 mm of the gap between the cathodes indicated approximately 2% (≒0.05 mm / 3 mm × 100) deviation. A small field size (approximately 20 mm × 20 mm) was adopted in this study, and the position-dependent dose response was negligible. Using a large field size (approximately 120 mm × 120 mm) might require position-dependent correction.

We introduced DR_spot_ as the dose rate of the uHDR scanned carbon-ion beam. The DR_spot_ value can be obtained using the new dose monitor connected to the irradiation control system. Several dose-rate definitions for scanned particle beams have been proposed (e.g., ADR, dose-averaged dose-rate (DADR), and sliding window)^16^. The pencil beam scanning (PBS) dose rate was defined by Folkerts et al.^17^ to account for the time structure of the delivery of a PBS treatment plan. Currently, there is no consensus on the uHDR definition to predict the FLASH effect. In addition, measurements against several dose-rate definitions are not straightforward because of the associated time structure. The detectors (e.g., AMC) employed in the current clinical practice of particle therapy are not intended to be used to measure the dose rate but the accumulated dose. Thus, a measurable dose-rate definition using the simple time element would be beneficial. Considering the dose-rate in a single pulse is worthwhile because the definition is used to evaluate the FLASH effect, although the radio-frequency (RF) electric field is too fast to measure the signals from the dose monitor. The accelerated particles in the synchrotron would be approximated as a pulsed beam with the same period (MHz) as the RF electric field. The dose-rate in a single pulse can be calculated at 1.3 × 10^4^ Gy/s using the parameters tabulated in Table 2. The nomenclatures in the table follows to the paper^18^. The beam parameters of the dose-rate in a single pulse and the total irradiation time are similar to the parameters reported previously^19^ in which a 10-Gy uHDR whole-brain irradiation using synchrotron generated X-rays did not induce memory deficit. This characterization could quantitatively verify uHDR carbon-ion parameters, especially those generated from the synchrotron.

**Table 2:** Overview of the relevant beam parameters estimated using the synchrotron for the uHDR carbon-ion beam.

Number of pulses delivered per unit time (208.3 MeV/u)	6.1 MHz
Number of pulses	1.13 × 10^4^
Pulse width	65.60 ns
Time between pulses	98.40 ns
Total irradiation time from the beginning of the first delivered	1.86 ms
Total delivered dose	9.67 Gy
Dose in a single pulse	8.54 × 10^−4^ Gy
Mean dose-rate for a multi-pulse delivery	5.21 × 10^3^ Gy/s
Dose-rate in a single pulse	1.30 × 10^4^ Gy/s

The dose rate of the actual uHDR beam deviated because of the ripple of the beam intensity (Figs. 3 and 4d–f). However, the ADR-based dose-rate definition for scanned beams depends on the total irradiation time. When the field size is large, the dose rate can be reduced, although the same irradiation dose is applied to a point. The FLASH effect conditions that were postulated (e.g., 40 Gy/s)^20^ were not derived from scanning beams but from scattered beams that did not have a time structure over the irradiation field. When the field size is small (i.e., 20 mm × 20 mm), the ADR-based dose rate could be employed to approximate the dose rate derived from the scattered beam owing to the short total irradiation time. As DR_spot_ is calculated using the dose and irradiation time for each spot, DR_spot_ can be used as an indicator of the FLASH effect as a spot-specific dose-rate definition.

Despite previous studies^13,14^ wherein disposed spots were introduced not to use a rising region of the beam current to create the field used in the experiments due to the timer control for the spot dose, we did not employ the disposed spot, as the new dose monitor could control the spot dose based on the MU. Consequently, the scanned carbon-ion beam was close to that used in clinical applications. The irradiation time of the first spot was relatively long because of the rising region of the beam current (Fig. 4a–c). The long irradiation time can be shortened by applying the RF knockout to the beam before starting the beam irradiation, which increases the amplitude of the beam (i.e., the particle density near the separatrix is increased, speeding up the rise time at the start of the beam irradiation). The highest dose was 9.7 Gy, which can be irradiated at 208.3 MeV/u using the current irradiation system considering the amount of charge stored in the synchrotron. The shortage of the charge in the latter spots resulted in a long irradiation time at 9.7 Gy (Fig. 4c), accounting for the large relative deviation of DR_spot_ from the ADR (Fig. 4f). The shortage of the charge can be enhanced by increasing the RF knockout voltage to maintain the beam current via the feedback control of the current and the diameter of the synchrotron’s vacuum duct (i.e., acceptance) to increase the amount of the charge. The fluctuation (i.e., ripple) of the beam intensity at the low dose per spot could induce the fluctuation of DR_spot_ because the ripple of the beam is not averaged out in the shorter irradiation time for low dose per spot (Figs. 3a and 4d).

One concern is the corrected charge reduction due to two recombination effects: the initial and volume recombinations. The initial recombination occurs within the track, and the volume recombination occurs owing to the intertrack combination effects. The initial recombination is independent of the dose rate and has been experimentally evaluated for carbon-ion beams at a normal dose rate^21^. For uHDR irradiation, the volume recombination is more relevant. Baack et al. investigated the recombination loss of different fill gases in ICs to achieve accurate dose monitoring^22^ and found that at high intensities, the He/CO_2_ mixtures enable the operation of the ICs at an electric field strength of 2 kV/cm or more, reducing the recombination to negligible levels at intensities larger than 3 × 10^10^ ^12^C-ions per second. The developed IC in this study was filled with air. The IC was manufactured by reducing the electrode spacing to increase the electric field strength and reduce the recombination effects, keeping an upper limit of the electric field strength (− 500 V/mm) for electric discharge. The dose monitor for clinical use has a 5-mm electrode spacing for an applied voltage of − 1200 V (i.e., − 240 V/mm), whereas the monitor for the uHDR beam has a 3-mm spacing for an applied voltage of − 1500 V (i.e., − 500 V/mm). Correction of the effects to compensate for the recombined charge carriers is not feasible because of the high-intensity fluctuations of the beams extracted from the synchrotron. In the synchrotron facility, a correction factor could not accurately account for the difference in the saturation effects during the high-intensity fluctuations of the spill extraction combined with the raster scanning of the beam, leading to an inhomogeneous dose distribution. However, the repeatability of the absolute-dose measurement was within 0.5%. The flatness of the dose profile was within 2.5%. Therefore, the developed IC could be employed for dose monitoring. Biological experiments will be conducted with the doses, dose-rates, and field size established here using the developed IC.

A new dose monitor for the uHDR-scanned carbon-ion irradiation was developed and characterized herein. The current monochromatic beam mode does not have a dose monitor and cannot control the beam in MUs. With the new dose monitor, the dose can be controlled in MUs and the dwell time, and the delivered dose can be monitored in the monochromatic beam mode. The new dose monitor also enabled the measurement of a dose rate for each spot defined as DR_spot_. To the best of our knowledge, no study has been conducted on the measured dose rates of each spot at an uHDR for scanned carbon-ion beams. The dose monitor successfully controlled the uHDR beams from low to high dose levels at an uHDR with the field size and dosimetric characteristics (Fig. 5 and Table 1), which are essential for further performing advanced FLASH research with complexly controlled scanned carbon-ion beams. The developed IC could be employed for the dose range used in the experiments with the uHDR beams. For clinical applications, it is necessary to ensure linearity over a wider dynamic range, for example, in the conventional dose rate range (< 33.7 MU/s corresponding to 6.4 Gy/s). Although the circuit constants used herein were selected for the beam current range in the uHDR region, it may be necessary to create a circuit that is capable of switching the constants according to the dose rate range or to increase the upper limit of the pulse rate that can be transmitted for the treatment machine (< 2,000,000 pulse/MU). Otherwise, linearity in the low dose rate range would be lost or the accuracy of MU input in the irradiation system would decrease. Clinical treatment plans with scanned beams include multiple energy layers. DR_spot_ could also be applied to defining the dose rate of a spot in each energy layer owing to the spot-specific dose-rate definition. Our current uHDR irradiation system does not have a spot position monitor (SPM). A SPM for uHDR should be developed for accurate monitoring and control of spot positions while the good flatness observed in this study without the SPM is due to the highly precise scanning system. In addition, although a cyclotron-based FLASH-radiotherapy (FLASH-RT) has been conceptualized, a synchrotron-based FLASH-RT strategy needs to be established^23^. Developments to irradiate a target in three or four (i.e., for moving targets) dimensions with synchrotron-based uHDR are mandatory, which will facilitate instantaneous volumetric irradiation (abbreviated as IVI).

## Conclusion

Herein, we developed and characterized a dose monitor for uHDR-scanned carbon-ion irradiation. With the dose monitor, the dose can be controlled in MUs and the dwell time, and the delivered dose can be monitored in the monochromatic beam mode which enables accurate and reliable dosimetry for successfully conducting experiments with the uHDR beam in the monochromatic beam mode. The DR_spot_ parameter was introduced for characterizing the scanned beam at the uHDR as a measurable simple indicator of the dose rate of the scanned carbon-ion beam. The lack of experimental validation of the dose rate can be achievable with the dose monitor. The dose monitor demonstrated the beam characteristics (i.e., dose, dose rate, and dose profile) of uHDR-scanned carbon-ion beams at several dose levels. The accurate and reliable dosimetry with the measurable simple dose rate contributes to establishing the synchrotron-based FLASH-RT and moving forward to translate the synchrotron-based FLASH-RT to clinical practice, which have never been established.

## Methods

### IC

**Figure 6:**
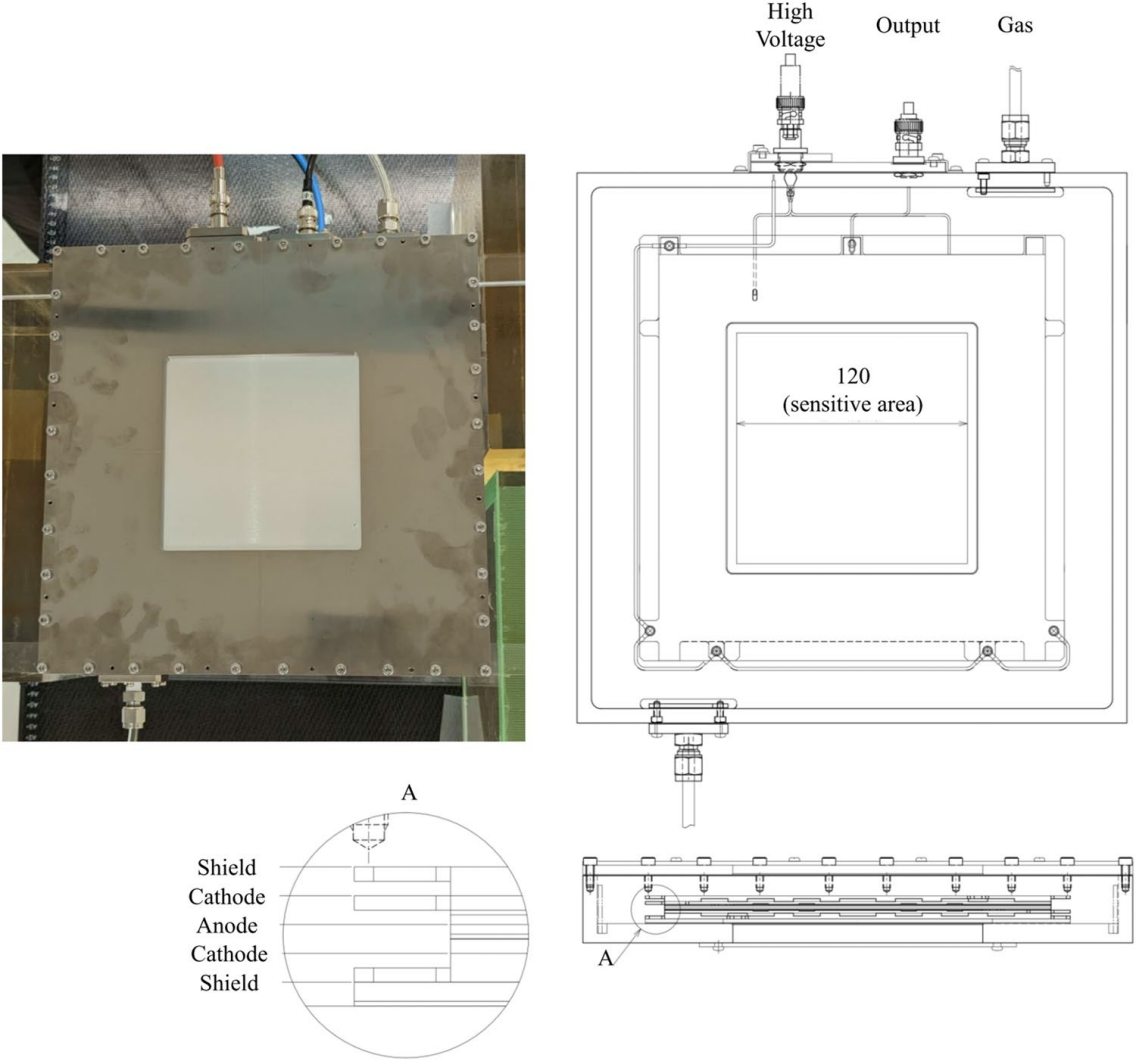
Schematic of the IC. The active volume of the large, plane-parallel IC is 120 mm × 120 mm × 3 mm.

A large plane-parallel IC with an active volume of 120 mm × 120 mm × 3 mm and a dedicated electrical circuit were developed (Hayashi-Repic, Tokyo, Japan) for the dose monitoring of uHDR beams. The electrode spacing of the IC was reduced to increase the electric field strength and reduce the recombination effects, which maintained an upper limit of the electric field strength (− 500 V/mm) for electric discharge. However, reducing the electrode spacing has disadvantages: manufacturing errors in the distance between electrodes can lead to measurement error, and noise generated by the oscillation of the electrodes can increase, causing a current to flow through the signal electrode, which results in the measurement error when the electrodes are shaken by sounds generated from the treatment machine due to mechanical motions. Because expanding the field size increases these issues, a smaller field size (120 mm × 120 mm) was selected compared to that of a clinically used dose monitor (200 mm × 200 mm). The IC comprises an anode at the center of the chamber surrounded by a cathode with a 3-mm gap between the cathodes (Fig. 6). The electrodes comprise aluminum foil with an average thickness of approximately 12 μm. The voltage (i.e., − 1500 V) applied to the IC was supplied using a high-voltage power supply module (Ortec 556, Oak Ridge, TN, USA). The chamber’s housing featured a gas inlet port and an outlet port, which enabled the utilization of arbitrary gas mixtures as the fill gas (Fig. 6) to investigate the difference in the ion collection efficiency due to the type of the fill gas, although the ports were not used in this study (i.e., the ambient air was used).

Figure S1 (Supplementary Material) shows a diagram of the signal processing chain. The IC is connected to the amplifier circuit, followed by the irradiation control system. The irradiation control system is controlled by the input pulse generated from the amplifier circuit. The amplifier circuit allows a maximum input current of − 50 μA because a relatively high ionization current is delivered using uHDR beams rather than that set for conventional dose rates (− 0.5 μA). The amplifier circuit was designed to satisfy the maximum operable voltage (10 V) and the maximum countable value of the pulse counter (1 MHz) to control the irradiation dose.

### Characterization of the operational amplifier

Figure S2 shows a diagram of the measurement for the characterization of the operational amplifier. A function generator (33500B, Keysight Technologies, Westlake Village, CA, USA) was employed for the input voltage. Input voltages ranging from − 0.001 to − 5 V were converted into currents (− 0.01– − 50 μA) via the voltage-to-current converter (50 μA/5 V). The converted current was measured using an electrometer (6517B, Keithley Instruments, Cleveland, OH, USA). To confirm the linearity of the output frequency against the input current, the output frequency of the amplifier circuit was measured using a digital oscilloscope (TBS2000, Tektronix, Beaverton, OR, USA).

### Experimental setup and irradiation plan for the raster scanner

**Figure 7:**
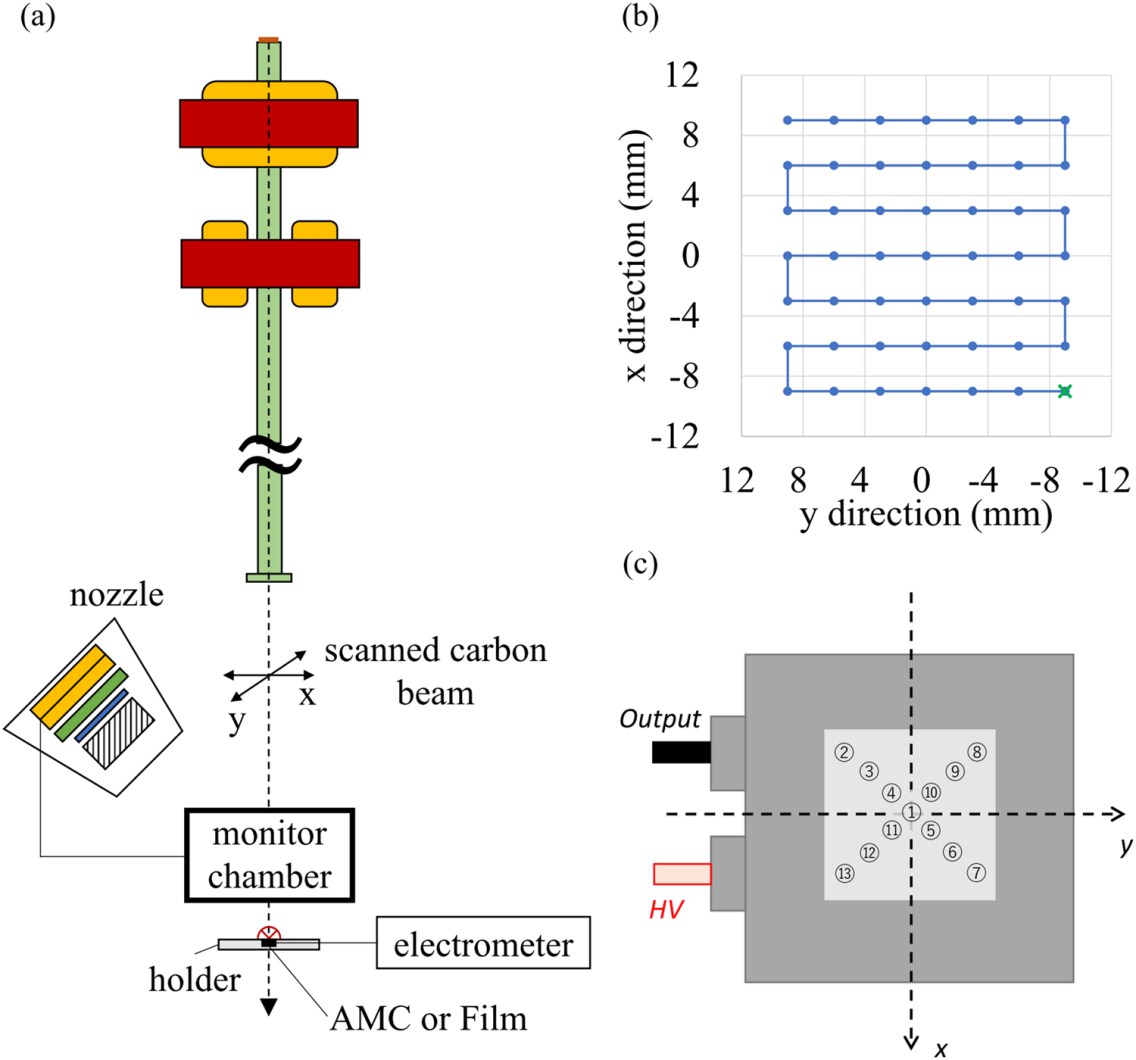
Setup for uHDR experiments. (**a**) Monochromatic beam mode in a treatment room. The carbon-ion beam did not pass through the nozzle. The advanced Markus chamber (AMC) and Gafchromic film were used to measure the absolute dose and the field size, respectively, at the plateau depth (**b**) Scanning pattern used in this study. The cross (in green) indicates the starting point of the scan. The carbon-ion beam was scanned once to create a field within the extraction time. The axes show the beam coordinates. (**c**) Measured positions for the spatial inhomogeneity of the dose response of the IC.

We performed uHDR measurements in the monochromatic beam mode^13,14^ using a 208.3-MeV/u carbon-ion beam extracted from the Heavy Ion Medical Accelerator in the Kansai (HIMAK) accelerator system^24^ at the Osaka Heavy Ion Therapy Center (OHITC)^13,14,25^ (Fig. 7a). The extraction RF power pattern during the extraction time was returned to allow higher charges to be deflected from their orbit into the extraction channel in the monochromatic beam mode. The accelerator could deliver > 1 × 10^9^ carbon ions per synchrotron cycle (spill). Ultrafast extraction of the entire charge in the spill was achieved within 100 ms (i.e., instead of the conventional 10,000–30,000 ms) by adjusting the amplitude of the extraction RF, typically requiring a power level several dozen times higher than that used in a routine radiotherapy operation. This resulted in considerably higher beam currents (i.e., typically 10 nA instead of 0.1 nA) being available for irradiation during one accelerator cycle. The beam current was measured using a digital oscilloscope (TBS2000, Tektronix, Beaverton, OR, USA) connected to the irradiation system^14,15^. The scanning speed was more than 7.5 cm/ms in the x direction and 15 cm/ms in the y direction. The current control precision of scanning magnets was <  ± 0.15% (corresponding to a beam position precision of less than ± 0.15 mm). The distance of the midpoint between the x- and y-scanning magnets to the isocenter was 6025 mm.

The pattern for the uHDR and conventional irradiations comprised 7 × 7 beam spot positions (Fig. 7b), with a grid spacing of 3 mm. Each spot position was irradiated only once (i.e., no repainting) to simplify the dose rate calculation. This irradiation pattern was employed for all measurements in this study.

The surface of the AMC (type 34,045, PTW-Freiburg GmbH, Freiburg, Germany) set to the center of a dedicated acryl holder (Accelerator Engineering Corporation, Chiba, Japan) was aligned to the isocenter with a room laser. The AMC was selected because the irradiated field size was small, and the highest possible electric field strength was employed in the operational voltage range.

Irradiation records involving MUs and the irradiation times of all spots were acquired from the irradiation system for each irradiation. The irradiation start time was the time the spot movement initiated (for the first spot, it started from the time of the RF knockout for the beam extraction). The irradiation stop time was the time of spot irradiation at which the irradiation at the planned MU finished, which was written in the SP file. When the MU of the spot reached the planned MU, the spot time counter was reset. The pulse output from the IC was connected to the pulse counter for the dose control circuit of the irradiation system (Fig. 7a), indicating that irradiation records are obtained from the HyBeat Heavy-ion Therapy System (Figure S1).

### Characterization of the IC

For the measurement of the saturation curve of the ion collection efficiency, the AMC connected to UNIDOS^webline^ (PTW-Freiburg GmbH, Freiburg, Germany) was used. The AMC was operated at 400 V, which resulted in a recombination factor of < 1%^14^. To test the recombination behavior of the IC, the saturation curves of the ion collection efficiency with electrode voltages in the range of − 100 V– − 1500 V were measured under uHDR conditions.

The dose linearity of the IC was measured by changing the dose from 9.8 to 65.1 MU (from 1.8 to 12.3 Gy). AMC readings (nC) were plotted against the corresponding values in MUs.

The dose response inhomogeneity of the IC was investigated. Dose responses were measured at thirteen positions (Fig. 7c): ① (0 mm, 0 mm), ② (− 45 mm, − 45 mm), ③ (− 30 mm, − 30 mm), ④ (− 15 mm, − 15 mm), ⑤ (15 mm, 15 mm), ⑥ (30 mm, 30 mm), ⑦ (45 mm, 45 mm), ⑧ (− 45 mm, 45 mm), ⑨ (− 30 mm, 30 mm), ⑩ (− 15 mm, 15 mm), ⑪ (15 mm, − 15 mm), ⑫ (30 mm, − 30 mm), and ⑬ (45 mm, − 45 mm). The IC was manually moved to each position while keeping the AMC centered on the beam. AMC readings were normalized based on ① (0 mm, 0 mm). The dose response inhomogeneity of the IC was plotted as a function of distance from ① (0 mm, 0 mm). The negative sign was used for the distance when the coordinate of the x axis was negative.

### Applications of dose monitoring and dosimetry

Redundant measurements with radiochromic films (Gafchromic film, EBT3; International Specialty Products, Wayne, NJ, USA) were performed, followed by scanning using an EPSON DC-G20000 flatbed scanner (J331B, Epson Seiko Corporation, Nagano, Japan) at 150 dpi to verify the consistency of uHDR distributions in the lateral dose profile. The radiochromic films demonstrated no dose-rate dependency^26^. The ImageJ software^27^ (version 1.53k, National Institutes of Health, Bethesda, MA, USA) was employed to extract the profiles. The flatness was defined by $\left( {d{\text{max}} - d{\text{min}}} \right) / \left( {d{\text{max}} + d{\text{min}}} \right)$ and calculated within ± 8 mm in the field.

Absolute dosimetry was conducted using the AMC with the same equipment as that employed for the measurement of the saturation curve of the ion collection efficiency. Three different doses were measured five times, and the average values and standard deviations of the measured doses were calculated. The expanded uncertainties (k = 2) of the measured doses varied by 1%–4% depending on the irradiation dose.

Several definitions of the dose rate have been proposed (e.g., DADR and sliding window)^16^. In this study, the dose rate was calculated as the total delivered dose divided by the total delivery time, referred to as the ADR^28^, calculated as follows:

1 $$\begin{array}{*{20}c} {ADR = \frac{{Dose_{AMC} }}{{\mathop \sum \nolimits_{i} t_{i} }},} \\ \end{array}$$

where *Dose*_*AMC*_ and *t*_*i*_ are the absolute dose delivered for the full field measured by the AMC and the irradiation time recorded by the irradiation system, respectively. The parameter *i* indicates the spot number. The dose rate of each spot can be calculated because the IC was connected to the time counter of the irradiation system, which could record MUs and the irradiation time. In conjunction with the absolute dose in the AMC, we defined *DR*_*spot, i*_ as follows:

2 $$\begin{array}{*{20}c} {DR_{spot,i} = \frac{{Dose_{AMC} }}{MU} \times \frac{{MU_{i} }}{{t_{i} }},} \\ \end{array}$$

where *MU*_*i*_ is the value recorded by the irradiation system and *MU* is the value per spot preset in the plan file.

### Supplementary Information

Supplementary Information.

## Data Availability

The datasets used and/or analyzed during the current study are available from the corresponding author upon reasonable request.
