# Supplementary material for: Development and characterization of a dedicated dose monitor for ultrahigh-dose-rate scanned carbon-ion beams
Source: Sci Rep. 2024 May 21;14:11574. doi: 10.1038/s41598-024-62148-2 (PMC11109334; doi:10.1038/s41598-024-62148-2)
Supplement: Supplementary file 1 — Supplementary Information. [file 41598_2024_62148_MOESM1_ESM.docx]

**Supplementary Information**

Development and Characterization of a Dedicated Dose Monitor for Ultrahigh-Dose-Rate Scanned Carbon-Ion Beams

Masashi Yagi et al., 2023

This file contains Supplementary Figures S1-S2.


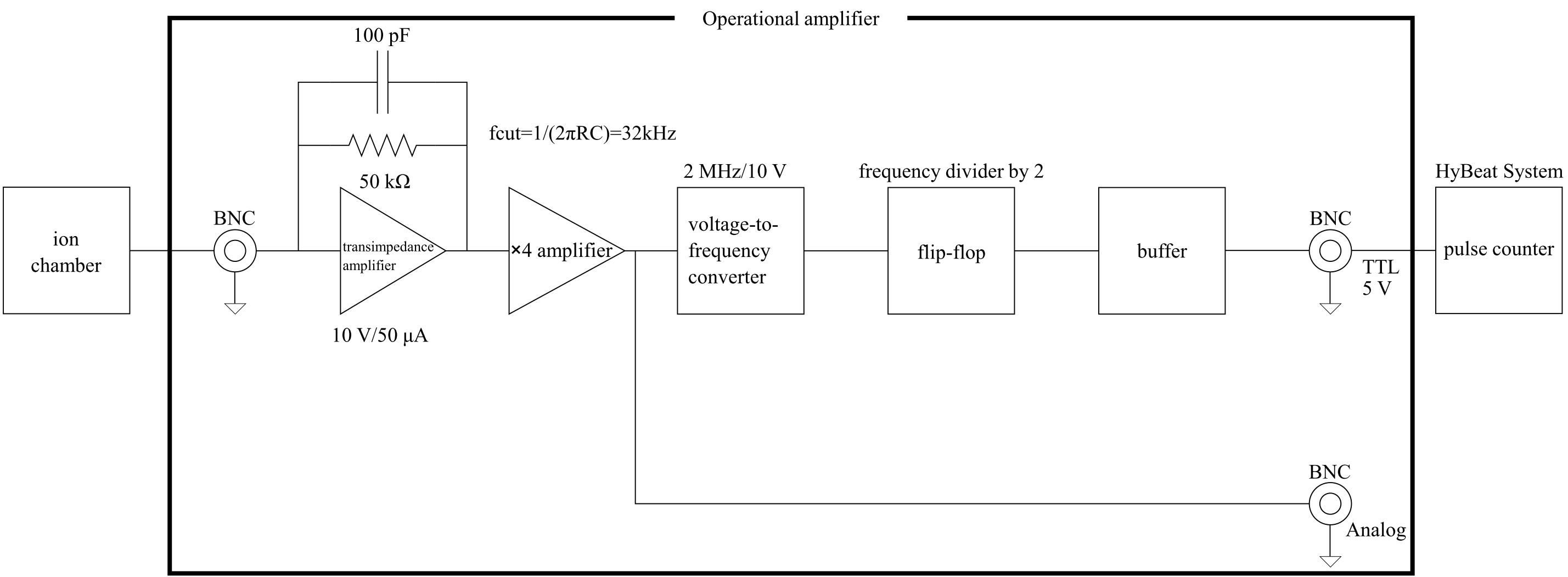


Supplementary Figure S1 Diagram of the dose monitor system. The ionization chamber is connected to the amplifier circuit, followed by the pulse counter of the irradiation control system.


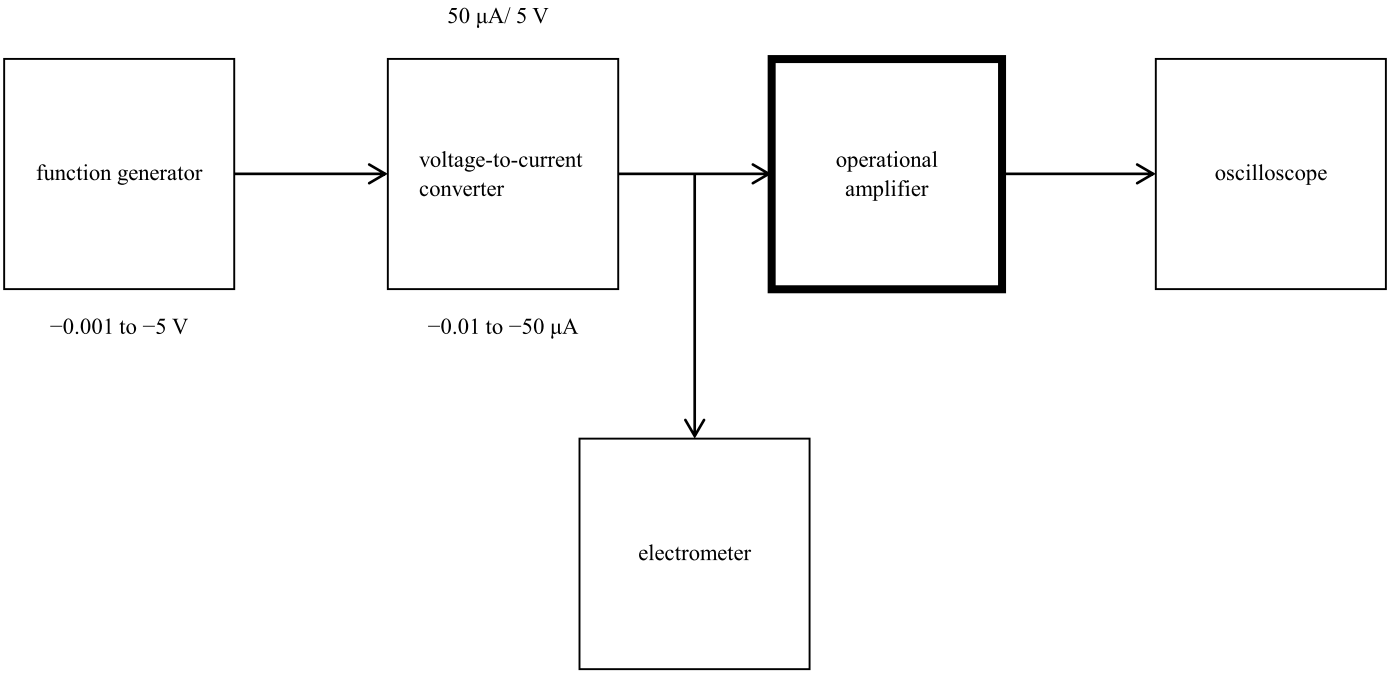


Supplementary Figure S2 Diagram of the measurement for the characterization of the operational amplifier. A function generator was used to supply the input voltage. The input voltage was converted into current using a voltage-to-current converter. The converted current was measured using an electrometer (middle of the diagram). To confirm the linearity of the output frequency against the input current, the output frequency of the amplifier circuit was measured using a digital oscilloscope.
